# A high security coding and anti-counterfeiting method based on the nonlinear magnetization response of superparamagnetic nanomaterials

**DOI:** 10.1038/s41598-024-65450-1

**Published:** 2024-07-04

**Authors:** Shi Bai, Yuxi Lin, Xiaoju Wang, Xiaodan Zhang, Takashi Yoshida, Xiaohan Yue

**Affiliations:** 1 Department of Information Engineering, Shenyang University of Technology, ShenYang, 110870 China; 2 Liaoning Vocational and Technical College of Economics, ShenYang, 110122 China; 3 Department of Electronic Engineering, Kyushu University, Fukuoka, 819-0395 Japan

**Keywords:** Superparamagnetic nanoparticles, Harmonic signal, Product code, Traceability, Biological techniques, Computational biology and bioinformatics, Nanoscience and technology

## Abstract

Traditional coding methods based on graphics and digital or magnetic labels have gradually decreased their anti-counterfeiting because of market popularity. This paper presents a new magnetic anti-counterfeiting coding method. This method uses a high-performance coding material, which, along with small changes to the material itself and the particle size of the superparamagnetic nanomaterials, results in a large difference in the nonlinear magnetization response. This method, which adopts 12-site coding and establishes a screening model by measuring the voltage amplitude of 12-site variables, can code different kinds of products, establishing long-term stable coding and decoding means. Through the anti-counterfeiting experiment of wine, the experiment results show that the authenticity of the coded products can be verified using the self-developed magnetic encoding and decoding system. The new coding technology can verify the anti-counterfeiting of 9000 products, with a single detection accuracy of 97% and a detection time of less than one minute. Moreover, this coding method completely depends on the production batch of the superparamagnetic nanomaterials, which is difficult to imitate, and it provides a new coding anti-counterfeiting technology for related industries with a wide range of potential applications.

## Introduction

The demand for high-value-added products has increased significantly because of societal progress and increased improvement of consumption level^1^. However, growth in demand has also led to a rise in the counterfeit market, which has become a major issue. According to a report by organization for Economic Co-operation and Development (OECD), the counterfeit and shoddy goods market is worth $540 billion US and accounts for 3.5% of global trade^2,3^. For example, the sale of counterfeit vitamins has resulted in severe bleeding disorders^4^, while fake French wine has disrupted the market^5^, even renowned products like China’s West Lake Longjing tea are frequently imitated^6^. These incidents have seriously damaged the reputation of China's famous products, violated the legitimate rights and interests of enterprises, endangered the survival and development of enterprises, but also harmed farmers, seriously affect agricultural production, to the vast number of consumers suffered physical, economic, spiritual multiple injuries, etc.^7^ Therefore, to combat the problem of counterfeiting, product anti-counterfeiting is necessary to ensure legitimacy and improve market transparency.

Traditional product anti-counterfeiting technology mainly includes Quick Response (QR) code technology, tracing by stable isotope effects, and tracing by spectral methods. QR code technology represents text and numerical information using geometric shapes, and compacts information like text, photos, and websites, into geometric squares using coding algorithms. Users can decode QR codes using a camera or decoding software to see the coded information. Although the generation of QR codes is unique, they can be falsified in large numbers using simple printing equipment. Moreover, some counterfeiters can fake both the query link and relevant data, thereby transferring consumers who scan the fake QR code label to a fake verification page, where the fake data are compared in the verification page and the identification results are difficult to detect. Because many QR code anti-counterfeiting labels only support one valid query. Hence, this inquiry mechanism cannot conduct pre-sale inquiries on goods, solve the problem of counterfeiting, or meet the requirements of market supervision^8–10^.

The use of stable isotope effects as a method of identifying organisms is based on stable isotope differences between organisms. However, stable isotope preparation and application are expensive and require specialized knowledge and equipment. Moreover, someone with access to the correct isotope information could try to copy the information and apply it to a counterfeit product, which reduces the technology’s credibility. Although stable isotopes can be detected by Isotope Ratio Mass Spectrometer, this requires specialized equipment and skills, which might limit the average user’s ability to determine a product’s authenticity. To apply stable isotope technology, it is necessary to add isotope labels to the product, which may affect the performance, appearance, or other aspects of the product. Therefore, for some products, especially high-value-added products or products that need to maintain their original quality, the use of stable isotope technology has limitations^11–14^.

Although spectroscopy can be used to quantitatively analyze different samples, including gases, liquids, homogenates, and powders, it requires the samples to be in a specific state and form. For example, solid samples need to be ground or dissolved. If sample requirements are not met, the spectrum’s accuracy and reliability may be affected. In complex samples, different substances may produce spectral features that interfere with each other, leading to erroneous results. The concentration of some analytes may be below the detection limit of spectroscopic instruments, especially in the analysis of trace components or trace elements. In some cases, spectroscopic methods may not have sufficient sensitivity. Spectral data often contain large amounts of information that require expertise to interpret and analyze. High-quality spectroscopy equipment are expensive and require highly skilled personnel to operate and maintain, which increases the cost and technical threshold of implementing spectral analysis^15–17^. Therefore, current methods of sample anti-counterfeiting analysis cannot meet the need for rapid anti-counterfeiting traceability of agricultural and high-value-added products.

### How can we design a new anti-counterfeiting method

A new anti-counterfeiting method uses mixed high and low-frequency odd and even harmonic signals to detect odd and even harmonic signals from various types of magnetic nanoparticles (MNPs) and magnetic response signals in solutions of mixed MNPs at different concentrations^18,19^. It also treats the magnetic response signal of MNPs that are functionalized via surface coating as a variable. These variables detect the corresponding voltage amplitude, thus establishing a screening model to obtain the coded data for identifying different kinds of products. To facilitate product anti-counterfeiting and traceability, a product coding database is established based on the obtained coding data, using a self-developed automatic coding and decoding detection system. The system verifies the feasibility of anti-counterfeiting and tracing the products. Currently, the system supports up to 12 coded data sites and it can identify up to 9000 products.

This paper explores the use of MNPs^20^ and harmonic detection^21^ for product anti-counterfeiting, because they are considered secure and stable, MNPs are suitable anti-counterfeiting coding materials, future advances may increase the number of coded data sites, enabling coding for tens of thousands of products. Therefore, the proposed method has the potential to support nationwide anti-counterfeiting traceability, particularly for agricultural products and other high-value-added products, the detection method uses low-cost MNPs, and the developed magnetic encoding and decoding system is user-friendly and does not require training, the entire detection process can be completed in less than one minute per product.

### Contributions to this article

#### New coding theory

The paper describes a new anti-counterfeiting method that utilizes the key physical parameters of superparamagnetic nanoparticles nanomaterials and their nonlinear magnetization response characteristics. The method utilizes single magnetic domain size variation, single magnetic domain magnetic moment, and hydrodynamic diameter, and the environmental solution of the MNPs significantly affects their rich harmonic response signals. Hence, they can generate a series of rich and unique password information and implement specific coding and decoding methods for product anti-counterfeiting.

#### System framework

The paper presents a comprehensive framework for an anti-counterfeiting system specifically designed for high-value-added products. This framework incorporates various components, such as anti-counterfeiting materials, product marking, anti-counterfeiting technology, product anti-counterfeiting systems, and coding and decoding identification.

## Materials and methods

### Superparamagnetic nanoparticles materials

#### Feature 1

The size of MNPs ranges from 5 nm to 10 µm^22^, to ensure their safety in the human body, small MNPs with diameters of less than 50 nm are commonly modified with hydrophilic flexible polymers like Polyethylene glycol (PEG) and dextran, which make them water-soluble and biocompatible. Polymer modifications confer the MNPs with various benefits, such as long-term storage stability, protection against environmental factors, improved dispersion, and enhanced manipulability. Their preparation process inevitably causes each batch of MNP materials to differ in magnetic core particle size or hydrodynamic particle size and Polymer dispersity index (PDI) dispersion. Hence, it is difficult even for manufacturers to imitate MNPs with the same nonlinear magnetization response, MNPs embody extremely high coding security.

#### Feature 2

Although the hysteresis of MNPs resembles that of a normal magnet, its magnetization response is completely different and it shows a remarkable nonlinear magnetization response characteristic dominated by the Langevin function. This nonlinear magnetization response is influenced by superparamagnetic nanoparticles nanomaterials and their molecular structure, as a special magnetic response force interval, the nonlinear magnetization characteristics of the solution are significantly affected by its environment, hydrodynamic outer diameter, magnetic core particle size, and MNPs shape. Moreover, nonlinear magnetization produces a very rich harmonic signal in addition to the fundamental wave, often the noisiest signal, which is usually discarded. For example, if Direct Current (DC) bias excitation is not applied, the odd harmonic signal (3, 5, 7, and 9) is generated, and even harmonic signals (2, 4, 6, and 8) can be generated when a DC bias excitation is applied. These signals can be saturated at a high magnetic field strength. Because of the relaxation time described below, real and imaginary part responses have different component proportions, depending on the conditions. This series of magnetized harmonic signals from each harmonic can provide a lot of coding information for MNPs. The coded information, with small changes, such as a change in the solution’s viscosity or a particle size bias of 2 nm, produces a significant difference in magnetization response. Therefore, multi-bit data can be coded based on the relevant characteristics.

#### Feature 3

When compared with other magnetic marker materials, MNPs exhibit a greater magnetization response, which makes them highly suitable for eigenvalue extraction. A greater magnetization response characteristic makes MNPs ideal for archival storage purposes. Moreover, MNPs possess excellent physical stability, which ensures high sensitivity and analysis accuracy, while expanding detection range. Hence, MNPs with superparamagnetic properties are chosen as marker materials for product anti-counterfeiting testing. The unique properties of MNPs make them promising materials for anti-counterfeiting and traceability applications, and they offer reliable code material for combatting counterfeiting in various products.

#### Relaxation time

The relaxation phenomenon is a dynamic response property of MNPs. At the microscopic level, the structural changes of MNPs can be reflected in macroscopic magnetic signals by changes in relaxation time. In MNP sample solutions, the Neal^23^ relaxation time is commonly expressed:

1 $${\tau }_{N}={\tau }_{0}\frac{\sqrt{\pi }}{2}\frac{1}{\sqrt{\sigma }}exp(\sigma )$$

2 $$\sigma =\frac{E}{{K}_{B}T}$$

3 $$E=\frac{\pi }{6}{d}_{c}^{3}K$$

In the equation, the characteristic time, is usually taken as ${\uptau }_{0}={10}^{-9}\text{s}$, ${d}_{c}$ is the inner diameter of the magnetic core, and K is the magnetic anisotropy constant.

Brownian^24^ relaxation time is commonly expressed:

4 $$\tau_{B} = \frac{{3\eta V_{H} }}{{K_{B} T}}$$

where $\upeta$ is the viscosity of the carrier solution (taking 1 mPa of pure water), *V*_*H*_ is the hydrodynamic volume of surface-modified MNPs:

5 $$V_{H} = \frac{{\pi d_{H}^{3} }}{6}$$

*d*_*H*_ is the hydrodynamic diameter of surface-modified MNPs, and *K*_*B*_*T* is the thermal energy (*K*_*B*_: Boltzmann constant, *T*: absolute temperature). The characteristic time^25^, *τ*_*eff*_, is the effective relaxation time and is given by the following equations:

6 $$\tau_{{{\text{eff}}}} = \frac{{\tau_{B} .\tau_{N} }}{{\tau_{B} + \tau_{N} }}$$

7 $$f = \frac{1}{{2\pi \tau_{eff} }}$$

The relaxation time determines how quickly MNPs return to their initial state after being magnetized. A smaller relaxation time implies faster recovery, which enables the MNPs to exhibit a more rapid response to high- and low-frequency AC excitation signals. Additionally, MNPs with shorter relaxation times can maintain their magnetization strength to a certain extent, which enhances their overall performance in various applications.

**Figure 1:**
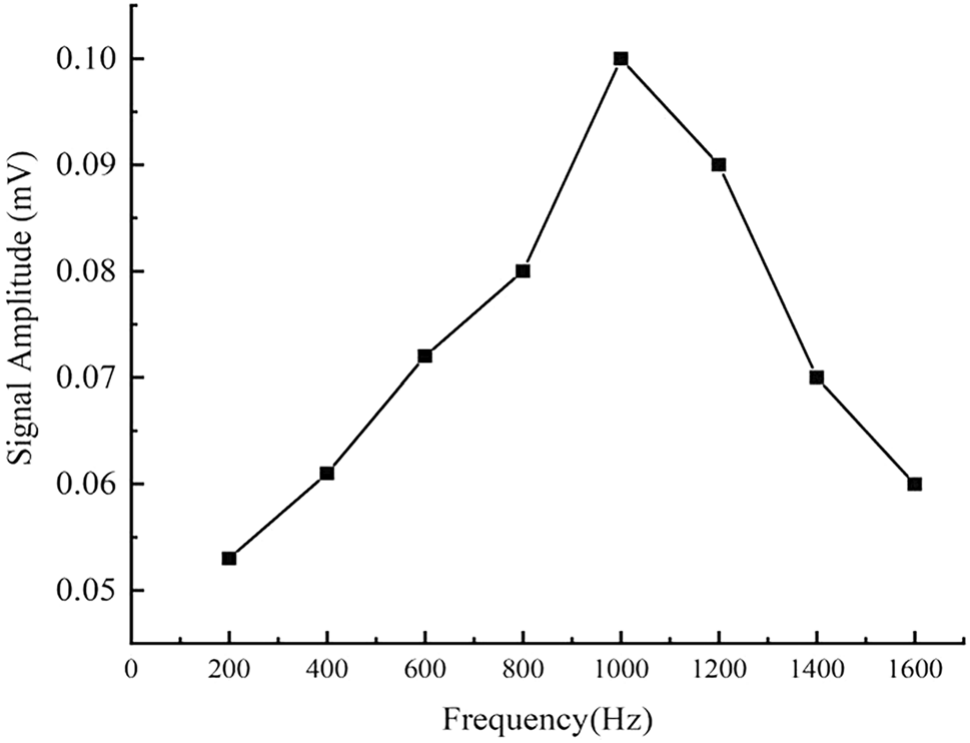
The relationship between signal amplitude and magnetic field frequency.

Due to the rich magnetic response signals of its MNPs, an MNP sample from Nanoeast (Nanjing Biotechnology Co., Ltd., China) (inner diameter: 30 nm) was selected, and 0.1 mL of the sample was diluted with 0.4 mL of PBS. Next, 0.1 mL of the diluted sample was transferred into a new sample bottle. The excitation magnetic field strength of the automatic coding and decoding detection system was set at millitesla (mT). As shown in Fig. 1, the signal peaked at around 1 kHz and then decreased rapidly with increasing frequency. To ensure that the signal could be obtained with small particle sizes and to improve the signal-to-noise ratio, subsequent experiments were done at 1 kHz as the actual excitation frequency. Figure 1 shows the third harmonic signal amplitude of the MNPs at different frequencies.

### Harmonic characteristics of MNPs

Because of the unique magnetic core structure and highly non-linear AC magnetization response of MNPs, it is difficult to effectively acquire their characteristic signals using conventional linear magnetization measurement methods^25^. To overcome this challenge, a dual-frequency AC excitation approach is employed, whereby MNPs are excited by simultaneously applying two alternating magnetic fields with different frequencies, so that higher-order harmonic signals are excited, and the odd harmonic signals are collected and analyzed. By analyzing these odd harmonic signals, the magnetization characteristics of MNPs can be measured more accurately. Moreover, because this method offers high signal stability and anti-interference capability, it is suitable for non-contact magnetization characterization during product anti-counterfeiting testing. A new anti-counterfeiting method demonstrates reliability and robustness in complex environments and provides effective technical support for product authentication and traceability. The magnetization characteristics of MNPs can be expressed as follows, using the Langevin function^26^.

8 $$L\left( \xi \right) = coth\xi - 1/\xi , \, \xi = \upmu_{0} m{\text{H}}_{0} /{\text{K}}_{{\text{B}}} {\text{T}}$$

where *m* is the magnetic moment of the magnetic core, K_B_ is the Boltzmann constant, T is the absolute temperature, and µ_0_ is the vacuum’s permeability. Under the action of an external magnetic field, the magnetization, *M,* of MNPs can be expressed^27^ as follows:

9 $$M\left( {\mu_{0} H({\text{t}})} \right) = M_{S} L\left( {\frac{{m_{0} \mu_{0} H(t)}}{{K_{B} T}}} \right)$$

where M_S_ is saturation magnetization, m_0_ is the magnetic moment, $\mu_{0}$ is vacuum permeability, *H *(*t*) is the applied magnetic field, K_B_ is the Boltzmann constant, T is the Kelvin temperature, and *L*( ) is the langevin function. An excitation field with an excitation strength, *H*(*t*), is applied externally to the MNPs and it can be expressed as follows:

10 $$H(t) = A_{1} \sin \left( {2\pi f_{1} t{}_{1} + \theta_{1} } \right) + A_{2} \sin \left( {2\pi f_{2} t_{2} + \theta_{2} } \right)$$

where $f_{1}$ is the frequency of the high-frequency excitation field, A_1_ is the initial amplitude of the high-frequency excitation field, *t*_*1*_ is the time, ${\theta }_{1}$ is phase angle, $f_{2}$ is the frequency of the low-frequency excitation field, A_2_ is the amplitude of the low-frequency excitation field, *t*_*2*_ is the time, and ${\theta }_{2}$ is the phase angle.

A non-linear magnetization response contains higher harmonics, which are integral multiples of the frequency of the external excitation field (i.e., k = 1, 2, 3…). By extending the right side of Eq. (8) in a Fourier series, the kth harmonic signal (odd harmonic signal) from the MNPs can be obtained^28^ using the following equation:

11 $$\begin{aligned} M & = M_{S} *L\left( {\frac{{M_{0} \mu_{0} H}}{{K_{B} T}}} \right) \approx \frac{{m_{0} \mu_{0} }}{{K_{B} T}} - \frac{1}{45}\left( {\frac{{m_{0} \mu_{0} }}{{K_{B} T}}} \right)^{3} H^{3} \\ & \quad + \frac{5}{756}\left( {\frac{{m_{0} \mu_{0} }}{{K_{B} T}}} \right)^{5} H^{5} - \frac{7}{2880}\left( {\frac{{m_{0} \mu_{0} }}{{K_{B} T}}} \right)^{7} H^{7} + \frac{7}{7920}\left( {\frac{{m_{0} \mu_{0} }}{{K_{B} T}}} \right)^{9} H^{9} \\ \end{aligned}$$

The real part and the imaginary part of alternating current (AC) susceptibility can be calculated using the Debye model and the Fokk–Planck function. The real part of AC magnetic susceptibility generally tends to decrease with increasing excitation frequency. When the excitation frequency is < 1 kHz, the real part of the AC magnetic susceptibility decreases slowly. When the excitation frequency exceeds 1 kHz, the real part of the exchange susceptibility rate begins to decline rapidly, while the virtual part peaks at particular frequency from 1 to 5 kHz. When the excitation frequency exceeds the peak frequency, the virtual part of the AC susceptibility begins to decrease. Because of the wide variation range of the real and imaginary parts, the product can be coded using the trends of the real and imaginary parts of the AC magnetic susceptibility.

**Figure 2:**
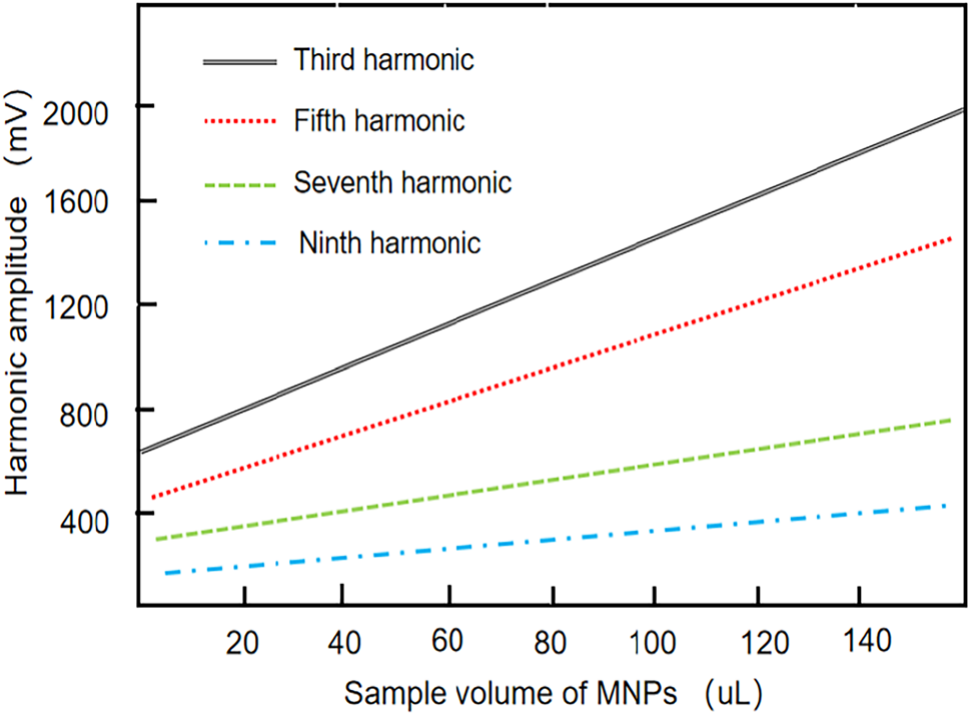
The harmonic characteristics of MNPs.

The analysis of the characteristics of the harmonic signals generated by MNPs under an excitation magnetic field of 7 mT^29^ was derived and verified using Eq. (11). Analysis of the harmonic signals generated by MNPs at an excitation magnetic field of 7 mT indicates that the amplitude of the odd harmonics increases with sample volume, while the amplitude of each odd harmonic decreases with an increase in the number of harmonic decompositions. This unique trend suggests that the magnetic response signal of higher harmonics is smaller than that of lower harmonics. MNPs can be used to code products based on their magnetic response properties. MNPs can exhibit unique magnetic signatures that are unaffected by changes in the external environment. Using the odd harmonic signals generated by MNPs, it is possible to create distinctive and specific coding data for different products, allowing for reliable and accurate product identification, even in varying conditions. Figure 2 shows the odd harmonic characteristics of MNPs.

Customized Nanoeast (inner diameter: 25 nm), Resovist (Fujifilm, Japan) (inner diameter: 30 nm) and Ocean (Nanotech USA) (inner diameter: 30 nm) magnetic MNPs were used as samples. Six different MNP solutions were prepared. Samples A and B are composed of MNPs from Nanoeast and carboxyl group, which are two samples with different hydrodynamic diametric diameter. Samples C and D are composed of MNPs from Resovist and carboxyl group, which are two samples with different hydrodynamic diametric diameter. Samples E and F are composed of MNPs from Ocean and dextran, which are two samples with different hydrodynamic diametric diameter.

**Figure 3:**
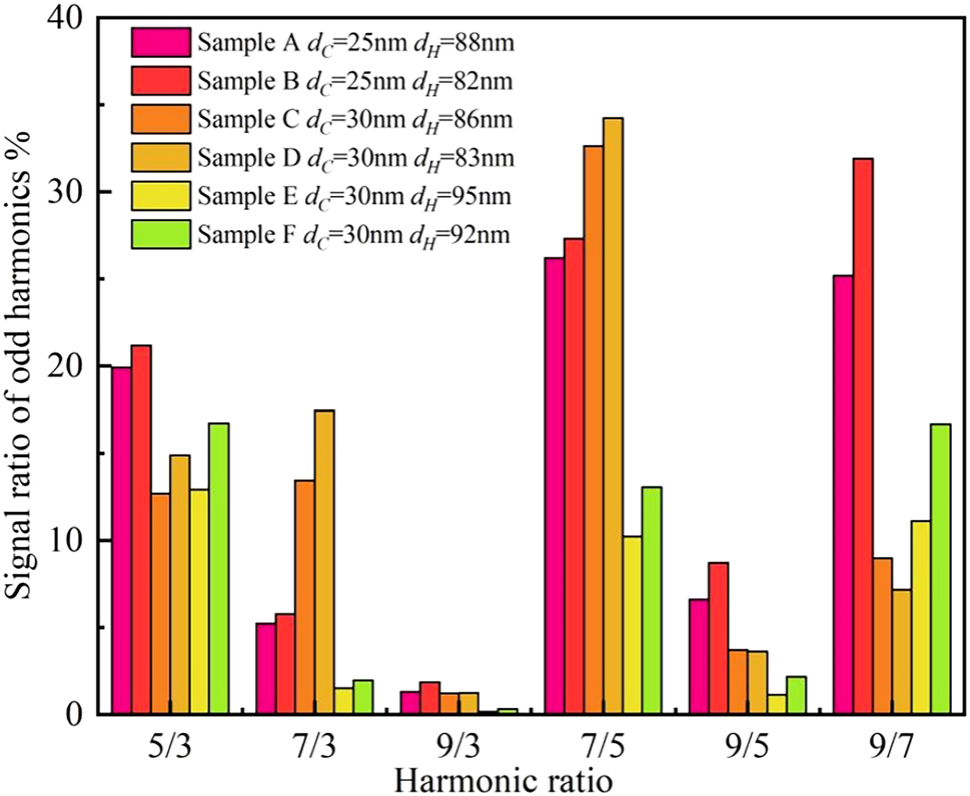
The harmonic signal ratio of MNPs.

**Figure 4:**
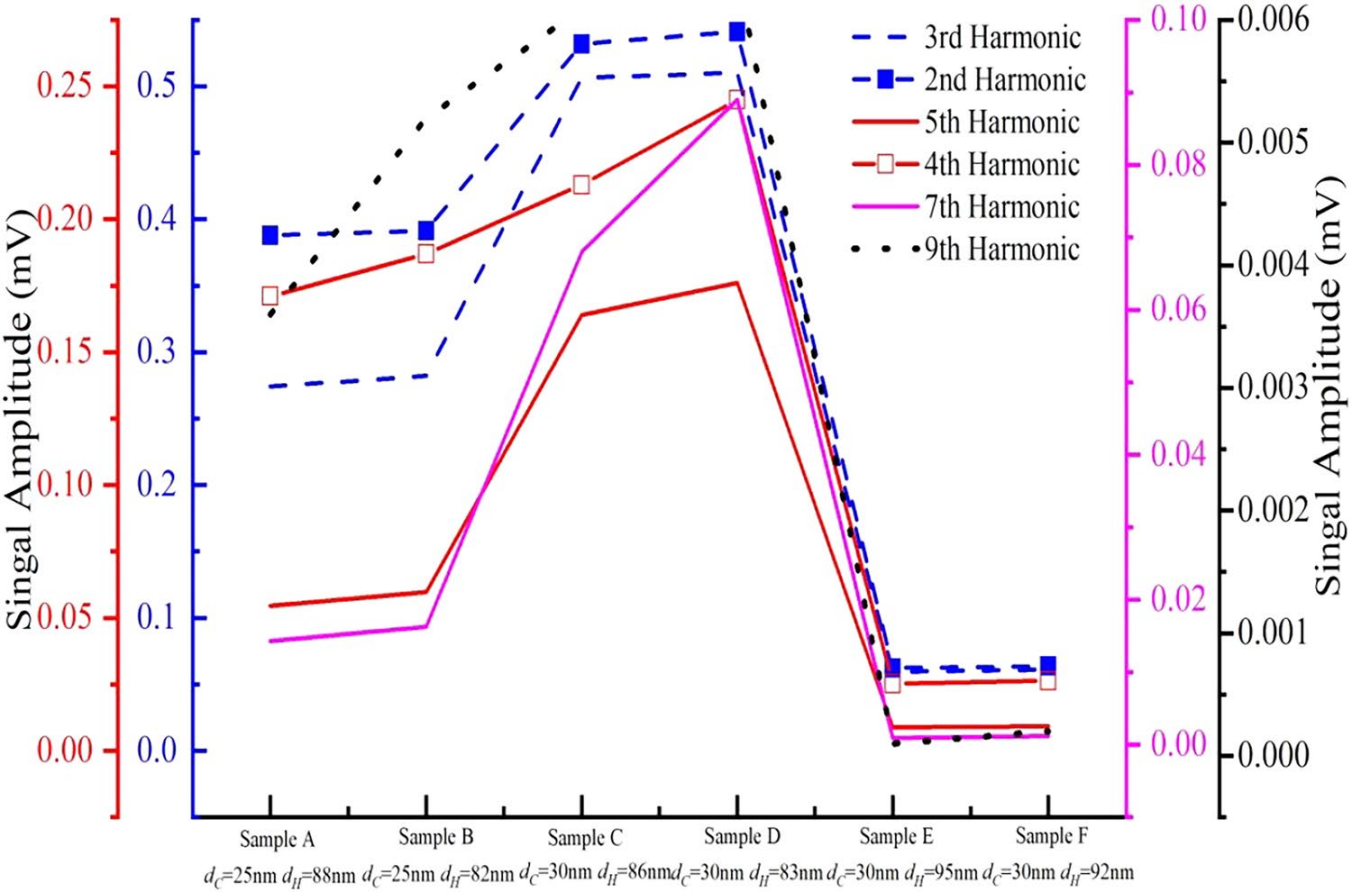
The harmonic signals of MNPs. The data show that under the same harmonic, the magnetic response signals of different types and concentrations of MNP solutions differ and the signals are unique.

Figures 3 and 4 show the harmonic signal ratios and the harmonic signals of the MNPs, respectively. The data in Figs. 3 and 4 show that under the same harmonics, different kinds of MNP solutions produce different signal values. Even for the same MNP solution, the harmonic signal amplitude differs because of small differences in magnetic particle sizes. Because relatively high MNP concentrations affect the spacing between ions in aqueous solution environments, a lower concentration environment causes ions to be more spaced out, allowing the random motions between ions to collide. The probability of van der Waals forces causing ions to unite increases at high concentrations and decreases at low concentrations. Relative to the actual response to ion dispersion, rather than the results measured by Particle size analyzer, different of the concentration will also affect the magnetic response signal. This difference provides a unique “fingerprint” for the product. Therefore, because of differences in various factors, including MNP kinds, coating layers, and magnetic core particle size, the MNP magnetic response behavior differs under a magnetic field, which provides a rich data source for coding.

### Particle size and material composition of MNPs

The coding method proposed in this paper uses MNPs as high performance coding materials. There are many kinds of magnetic materials, Fe_3_O_4_ is one of the most widely used, its particle size distribution is about 1–100 nm. When the particle size of MNPs is 30 nm, with the further reduction of particle size, MNPs exhibit superparamagnetic properties. It has strong magnetism under the external magnetic field, but the magnetism will disappear quickly after the exclusion of the magnetic field. MNPs with smaller particle size have faster magnetic response and stability, when the particle size of MNPs is larger than 30 nm, the response speed is slower but the magnetic response signal is stronger. Second, MNPs can be surface-modified, bring in a layer of polymer (e.g., polyvinyl alcohol, polyacrylic acid, gelatin, etc.). Hydrophilic groups or oleophilic groups (–CHO, –OH, –SH, –COOH, –NH_2_, etc.) or MNPs can be combined with proteins, enzymes, vitamins, DNA and other bioactive substances. These methods can regulate the chemical stability and dispersion of MNPs. The magnetic properties of MNPs can be regulated by different surface modification methods, which can provide rich coding information for MNPs material coding.

### The developed magnetic encoding and decoding system

**Figure 5:**
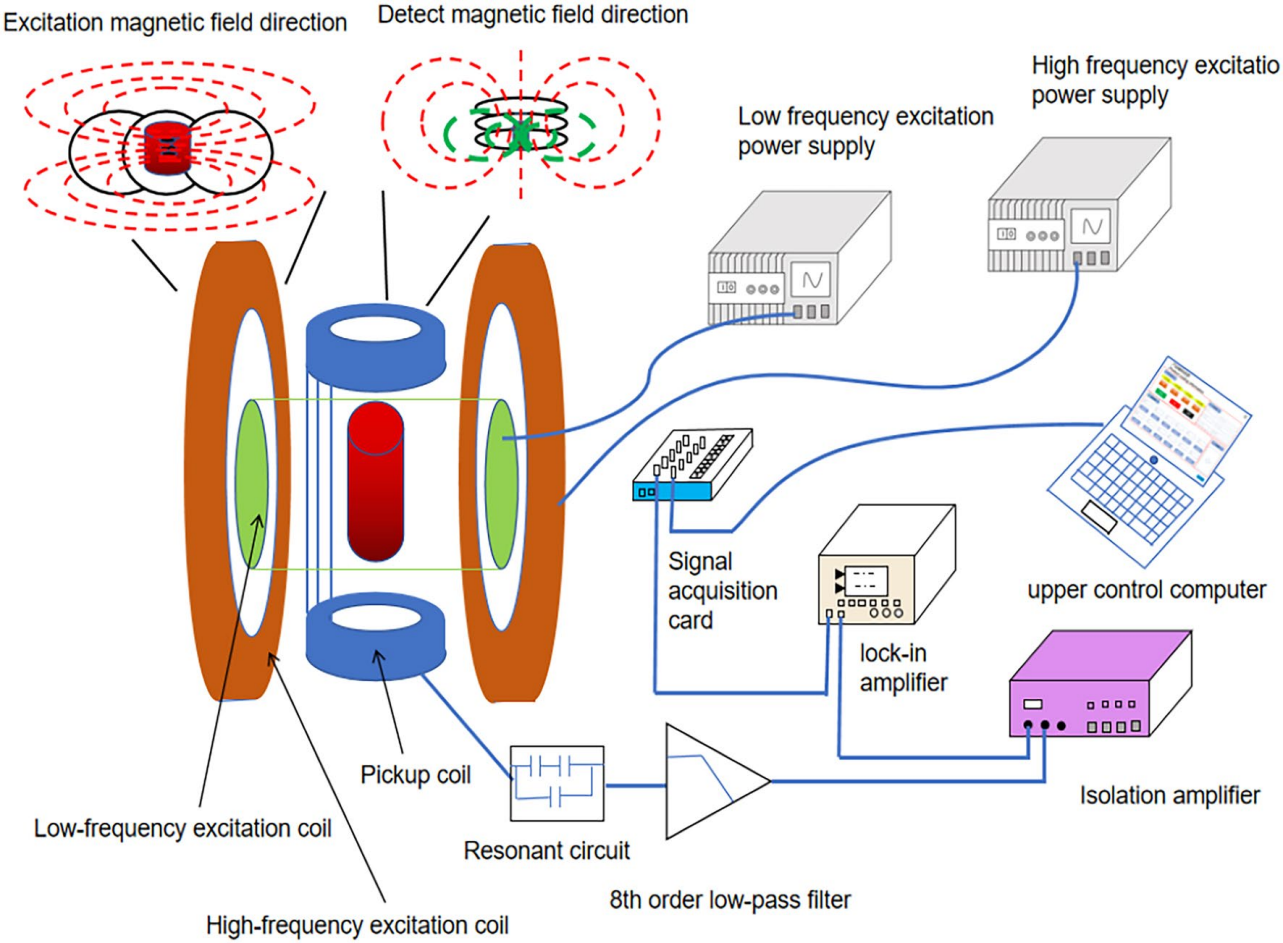
A schematic diagram of the developed magnetic encoding and decoding system.

The developed magnetic encoding and decoding system based on MNP materials has high sensitivity and reliability and can trace the whole process of high-value-added products. The system’s core parts are the pickup coil and excitation device, and the coded signal is processed and reconstructed by a signal processing unit and host computer unit. The differential pickup coil^26^ makes the excitation field to form a balanced state in the pickup coil, reduces fundamental frequency interference, and can detect weak magnetic nanoteslas nT-level signals. The system uses Helmhertz coils^30^ to generate a high-frequency magnetic field and a cylindrical coil to generate a low-frequency magnetic field, which plays a role in improving magnetic field intensity. Moreover, the upper computer unit can measure and reconstruct odd and even harmonic signals using in-house program, and a magnetic shield device is used to prevent interference from the environmental magnetic field. Figure 5 show a schematic diagram of the developed magnetic encoding and decoding system.

### Coding module

#### The coding principle

**Figure 6:**
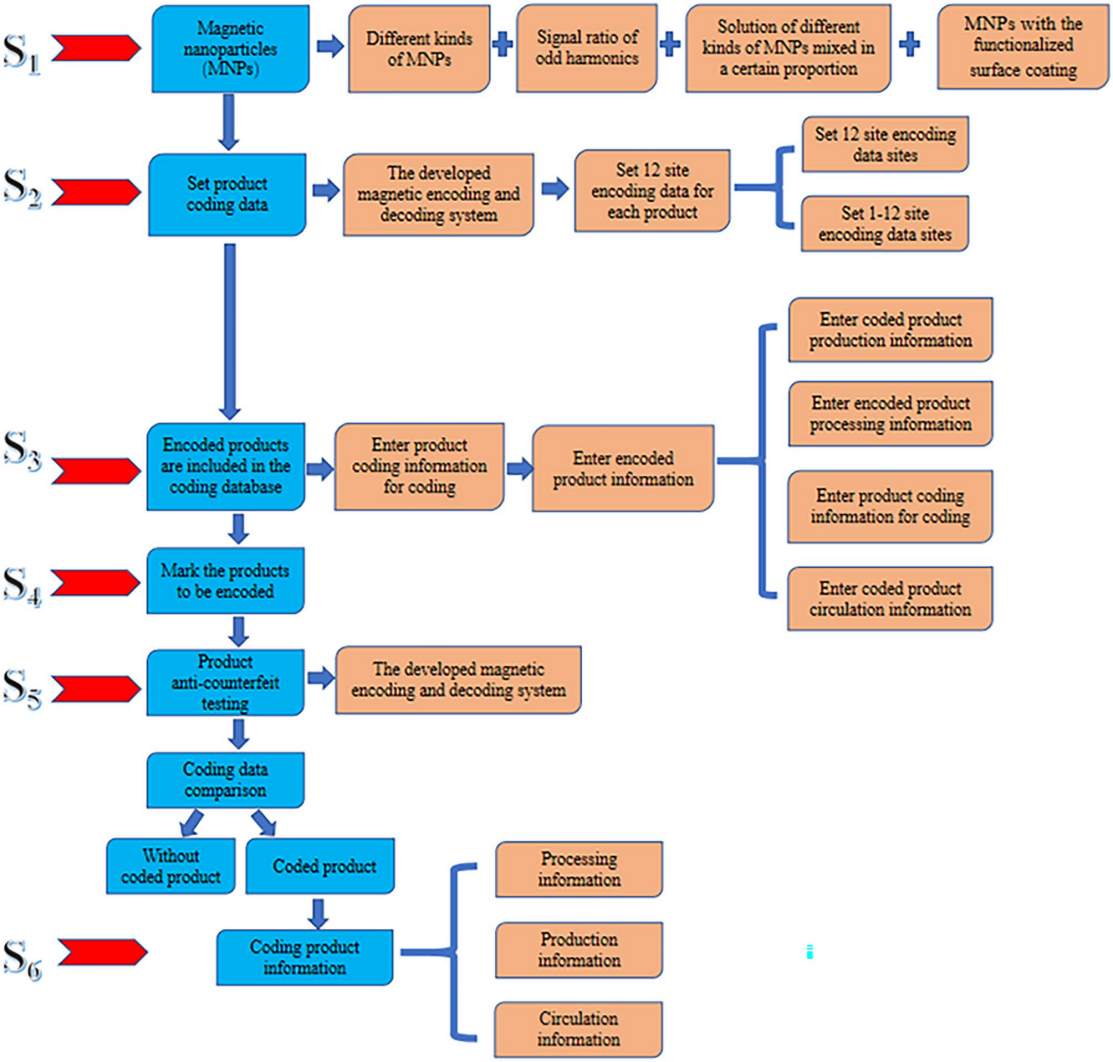
Flowchart of the coding principle. S_1_: MNP preparation. S_2_: carry out coding tests on products based on the coding principles. S_3_: enter coded data into the database. S_4_: carry out decoding tests according to decoding principles. S_5_: anti-counterfeiting tests.

Based on product anti-counterfeiting requirements, the harmonic voltage amplitude of different types of MNPs, the ratio of each harmonic voltage amplitude, and the harmonic voltage amplitude of the mixed solution of MNPs are used to determine whether the product is coded, based on the following coding principle. S_1_: Different kinds of MNPs are selected based on the demand and prepared into anti-counterfeiting labels. Various magnetic response properties are obtained by adjusting various MNP parameters, such as the coating layer, shape, particle size, and the type of target coupling protein of the MNPs. S_2_: Preparatory experiments are carried out on the products that need to be traced back to measure their harmonic voltage amplitude, the ratio of odd harmonic voltage amplitude, and the magnetic response signals of the mixed solution of MNPs. These data can be measured using the in-house automatic compilation code recognition system, with the results determining the value of each coding data site. To ensure each product has unique coding data, the number of coding sites can be set to 12 or several of the 12 coding data sites in the coding process. S_3_: The coded information for each product is stored in the product coding database, allowing for comparison during product anti-counterfeiting validation. S_4_: In product anti-counterfeiting involves measuring the harmonic voltage amplitude of various types of MNPs, the ratio of odd harmonic voltage amplitude, the harmonic voltage amplitude of MNPs with functionalized surface coating, and the harmonic voltage amplitude of mixed solutions of various types of MNPs. S_5_: These measurements are then compared with the data in the product coding database to establish each data site’s consistency with the corresponding data sites in the coding database. If deviation is detected, it is necessary to establish if it falls within an acceptable range and if it does, if the product passes anti-counterfeiting verification. However, a deviation that exceeds the acceptable range may indicate problematic or counterfeit products. Figure 6 shows the coding principle.

### Coding method

#### A mixed solution of different types of MNPs

When different types of MNPs or MNPs with different coatings are mixed at various ratios^31^ (1:1, 1:3, 1:6, and 1:9), they experience mutual gravitation and repulsive forces that cause them to agglomerate in the solution, agglomeration increases MNP particle size and changes their relaxation time, which affects the magnetic response signal generated by the mixed solution. Notably, the magnetic response signal of a mixed solution differs from that of a solution with two types of MNPs only. Mixing two types of MNP solutions at different proportions can generate different harmonic signals for product coding. Therefore, the harmonic signals of MNPs mixed with other MNP types or MNPs with different coatings can be used as a parameter for coding products.

#### MNPs with functionalized surface coating

The surface of the magnetic cores is coated with hydrophilic materials^32^ like oleic acid, which can prevent magnetic core aggregation and impart hydrophilic properties to the MNPs. Additionally, modifying the outer layer of the hydrophilic shell can achieve MNP binding to various functional target proteins or molecules using chemical bonds or biological proteins with coupling functions, this produces a range of MNPs with different harmonic signals. Particle size changes before and after coupling MNPs with the target protein affect the relaxation time of the MNPs and the magnetic response signal. Coupling the targeted proteins with MNPs generates unique magnetic response signals, and changing the target protein type achieves diverse MNP magnetic response signals. Hence, the harmonic signals of MNPs modified with functionalized surface coating can be used as coding parameters for product coding purposes.

#### Odd and even harmonic components

**Figure 7:**
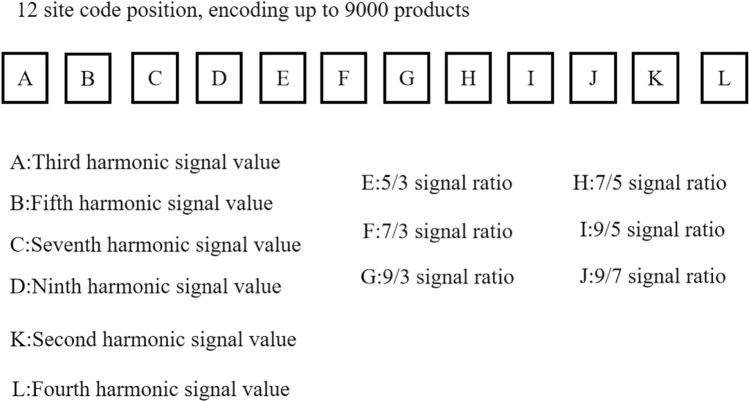
Code sites. The meaning of the coded data for each coding site is shown.

The study employed a coding approach based on the odd and even harmonic signals of the MNP solutions for product coding^33^. Specifically, the 2nd, 3rd, 4th, 5th, 7th, and 9th harmonic signals of the MNPs were used as coding data sites. Each harmonic signal exhibits unique magnetic response characteristics, ensuring coding distinctiveness. The odd harmonic signal ratio is calculated by dividing the amplitude of the 9th harmonic signal by the amplitude of the 3rd harmonic signal, resulting in the ratios, 9/3, 7/3, 5/3, 7/5, 9/5, and 9/7, measuring these signal ratios generates six independent coded data sites. Using this coding technique, we achieved reliable product coding using the magnetic response signals of the harmonic components of the MNPs as coding parameters. Figure 7 shows the code sites.

#### Screening model

To generate nonlinear magnetic response signals by magnetizing MNPs in the excitation device, which are collected by the pickup coil. Isolation amplifiers are used to filter and amplify signals to ensure data quality and reliability. The voltage amplitude of odd-order and even-order harmonic signal and the magnetic response signal of MNPs are obtained by using a phase-locked amplifier. The screening model was constructed with LabVIEW, the logic comparison module and the mathematical operation module were used to calculate the odd harmonic signal ratio, and the difference between the measured data and the database data was compared to determine its consistency. A threshold or tolerance range of ± 3% is set to determine whether the deviation between the measured data and the encoded database data is within an acceptable range. The logical judgment module of LabVIEW is used to make a logical comparison between the comparison result and the set threshold value. The results of logical judgment are integrated, and the screening model is realized by the logical structure of LabVIEW. According to the result of model judgment, the anti-counterfeiting verification of the product is carried out. If the measurement data is consistent with the coded database data and the deviation is within ± 3%, the product passes the verification; Otherwise, there may be problems or shoddy products that need further treatment or inspection. According to the verification results, the LabVIEW program gives the corresponding feedback information, displays the prompt of passing or failing the verification, and records the verification results for subsequent reference.

#### Decoding module

The system setup shown in Fig. 5 was used to construct the detection platform for the identification system to enable product coding detection and anti-counterfeiting. In the experiment, the MNP sample was magnetized using a mixing AC magnetic field (model: NF4610, NF4620), and the magnetic response signal of the MNPs was collected by a pickup coil. The magnetic signal was then converted into an electrical signal by a resonant circuit, an isolation amplifier (model: NF5325), and a phase-locked amplifier (model: NF5645). Electrical signals were then transferred to a personal computer for processing, analysis, and storage using LabVIEW software. The entire process of detecting the magnetic response signal takes less than a minute.

To validate the proposed method, determine its feasibility, and improve detection efficiency, magnetic field intensity was screened. Next, each coding method was tested to verify that the harmonic amplitudes generated using each method are different and that they do not correlate with each other. Finally, products were analyzed to detect their individual 12 coding data sites and the detected information was compared with the product code information in the anti-counterfeiting code database, which confirmed the system’s detection capability for coding anti-counterfeiting.

## Results and discussion

### Product code anti-counterfeiting analyses

#### Code site: different types of MNPs

The study used an automatic code detection system to determine the differences between the harmonic amplitudes of various types of MNPs. Four types of MNP samples (samples D, F, G, and H) with inner diameters of 30 nm were selected and 0.1 mL of each sample was diluted in 0.4 mL of PBS, followed by the transfer of 0.1 mL of the diluted samples to sample bottles. Next, the automatic coding and decoding detection system was used to apply an excitation magnetic field strength of 4 mT (frequency: 1 kHz) to each MNP sample and to measure the amplitude of the samples’ harmonic amplitudes. The experiment is repeated at least three times, and the average harmonic signal amplitudes of different MNP types can be used as encoded information. Table 1 shows the coding information of the different types of MNPs.

**Table 1:** The coding information of different types of MNPs.

MNP category	Harmonic order	Signal amplitude (mV)	Signal amplitude (mV)	Signal amplitude (mV)	Mean amplitude (mV)
Sample D (30 nm)	3	0.0999	0.1	0.1001	0.0925
Sample F (30 nm)	3	0.1375	0.1363	0.1365	0.1292
Sample G (25 nm)	3	0.2594	0.2588	0.2589	0.251
Sample H (30 nm)	3	0.335	0.331	0.332	0.3251

#### Code site: signal ratio of the odd harmonics

Sample D (0.1 mL) was diluted in 0.4 ml of PBS and 0.1 mL of the diluted sample was transferred into separate sample bottle. The automatic coding and decoding detection system was then used to apply an excitation magnetic field strength of 4 mT (frequency: 1 kHz) to the samples. The signal amplitude corresponding to each harmonic was then measured in each sample, and each experiment was repeated thrice to ensure data reliability. The average amplitude of the three measurements for each odd harmonic signal was calculated to obtain the ABCD-coded sites. Additionally, the ratios of different harmonic amplitudes, such as the 5th harmonic amplitude divided by the 3rd harmonic amplitude, were calculated to obtain the EFGHIJ-coded sites. ABCDEFGHIJ-coded sites were statistically analyzed. These analyses revealed no correlation between different harmonic amplitudes. Table 2 shows the coding information of the magnetic signal from a magnetic sphere under the 3rd, 5th, 7th, and 9th harmonics.

**Table 2:** The coding information of the magnetic signal from a magnetic sphere under the 3rd, 5th, 7th, and 9th harmonics.

MNPs	Harmonic order	Signal amplitude (mV)	Signal amplitude (mV)	Signal amplitude (mV)	Mean amplitude (mV)	Harmonic ratio		
Sample D (30 nm)	3	0.0999	0.1	0.1001	0.0925	5/3 7/3 9/3	7/5 9/5	9/7
	5	0.0432	0.0432	0.0432	0.0404	43.67%	51.49%	71.63%
	7	0.0224	0.0224	0.0224	0.0208	22.49%	36.88%	
	9	0.0156	0.0154	0.0155	0.0149	16.11%		

#### Code site: even harmonics signal

Sample D (0.1 mL) was diluted with 0.4mL PBS, followed by the transfer of 0.1 mL of the diluted sample to a separate sample bottle. Next, DC bias excitation was added to the automatic coding and decoding detection system to measure the harmonic amplitude corresponding to each harmonic for sample bottle. The experiment was repeated thrice, calculating the average amplitude of the three measurements of each even harmonic amplitude to obtain the KL-coded sites. This analysis showed no correlation between different harmonic amplitudes or between odd and even harmonic amplitudes Table 3 shows the coding information of the magnetic signal from a magnetic sphere under the 2nd and 4th harmonics.

**Table 3:** The coding information of the magnetic signal from a magnetic sphere under the 2nd and 4th harmonics.

MNPs	Harmonic order	Signal amplitude (mV)	Signal amplitude (mV)	Signal amplitude (mV)	Mean amplitude (mV)
Sample D (30 nm)	2	0.1132	0.113	0.1131	0.1131
	4	0.0511	0.0512	0.0518	0.0513

#### Code site: a solution of different types of MNPs mixed at 1:1, 1:3, 1:6, and 1:9 proportion

To determine the difference between mixed solutions and the signal values of the respective solutions of the two types of MNPs. Samples D and F (inner diameters: 30 nm) were selected and mixed at ratios of 1:1, 1:3, 1:6, and 1:9. The magnetic signal amplitudes of the mixed solutions were then measured at the 3^rd^ harmonic. This experiment was repeated thrice and the mean values were taken as coding sites. For each type of MNP, 0.1 mL of the sample was diluted in 0.4 mL of PBS. Next, 0.1 mL of the diluted sample D was transferred into four sample bottles, whereas 0.1, 0.3, 0.6, and 0.9 mL of the diluted sample F were transferred into separate sample bottles. The automatic coding and decoding detection system was then used to apply an excitation magnetic field strength of 4 mT (frequency: 1 kHz) to the MNP samples, and their harmonic signal amplitudes were measured using the system. This indicates that signal amplitudes do not significantly correlate between the mixed solutions and the signal amplitudes of the respective solutions of the two types of MNPs. Table 4 shows the coding information of the different kinds of mixed MNP solutions.

**Table 4:** The coding information for the mixed solutions of different types of MNPs.

Mixing ratio	Harmonic order	Signal amplitude (mV)	Signal amplitude (mV)	Signal amplitude (mV)	Mean amplitude (mV)
1:1	3	0.1549	0.1555	0.1545	0.1549
1:3	3	0.2748	0.2748	0.2748	0.2748
1:6	3	0.3999	0.3989	0.3990	0.3992
1:9	3	0.4322	0.4322	0.4322	0.4322

#### Code site: MNPs with the functionalized surface coating

The difference in the signal values of the MNP solutions modified with various chemical bonds or proteins was assessed. Sample D (inner diameter: 30 nm) MNPs were mixed with 0.9% saline or lipase, to obtain MNPs that produced different harmonic signals, and the magnetic signal amplitude of the two combined solutions was measured at the 3rd harmonic. The experiment was repeated thrice, calculating the average amplitude of the three measurements of harmonic amplitudes to obtain the coded sites. This analysis showed that there was a difference but no correlation between the signal amplitudes of the solutions of MNPs modified with various chemical bonds or proteins. Table 5 shows the coding information of the MNPs with functionalized surface coatings.

**Table 5:** The coding information of the MNPs with functionalized surface coating.

Types of coupling targeted materials	Harmonic order	Signal amplitude (mV)	Signal amplitude (mV)	Signal amplitude (mV)	Mean amplitude (mV)
0.9% physiological saline	3	0.4152	0.4157	0.415	0.4078
Lipase	3	0.5	0.4999	0.4996	0.497

### MNP stability analysis

**Figure 8:**
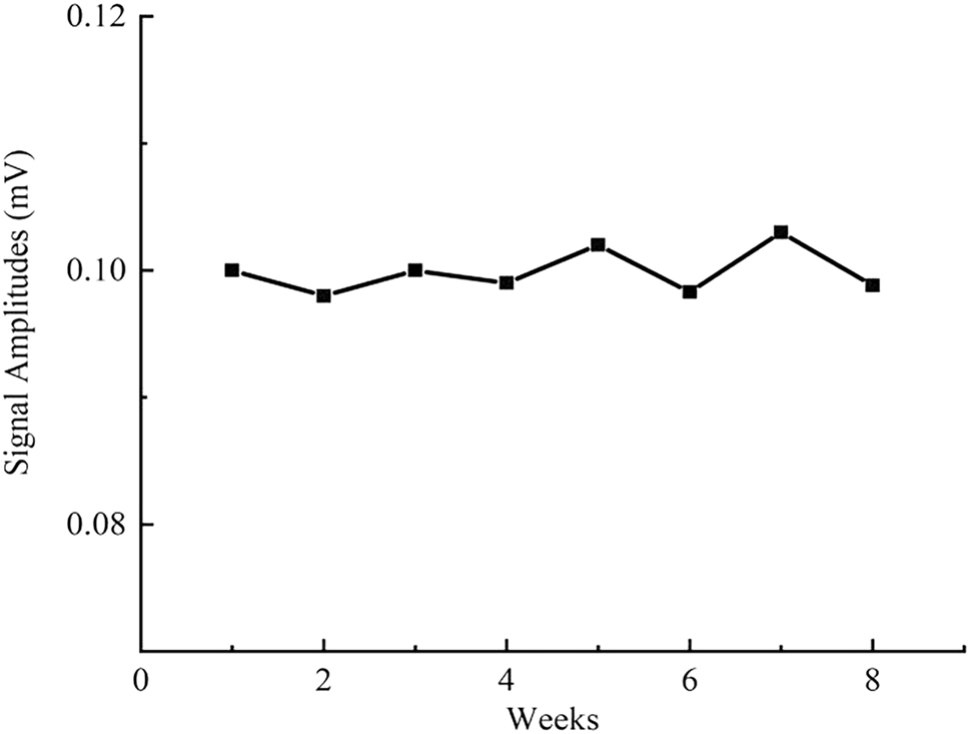
MNP stability analysis confirmed that the magnetic properties of the MNPs were stable and that they could be used as long-term anti-counterfeiting coding materials.

Sample D (0.1 mL) was diluted with 0.4mL PBS, followed by the transfer of 0.1 mL of the resulting solution to a sample vial and the addition of 10 µL of a Proclin300 (AbMole, USA) preservative, the magnetic signal amplitude is measured at the third harmonic. The experiments were repeated thrice a week for two months. The experimental results show that the signal amplitude ranged between 0.09 and 0.1 mV and the signal deviation range was within 0.02%, minimal MNP signal fluctuation over the two months of testing, the coded material can be used for long-term storage without significant degradation or changes in magnetic signal properties. Figure 8 shows the amplitude of 3rd harmonic signals during the MNP stability analysis.

**Figure 9:**
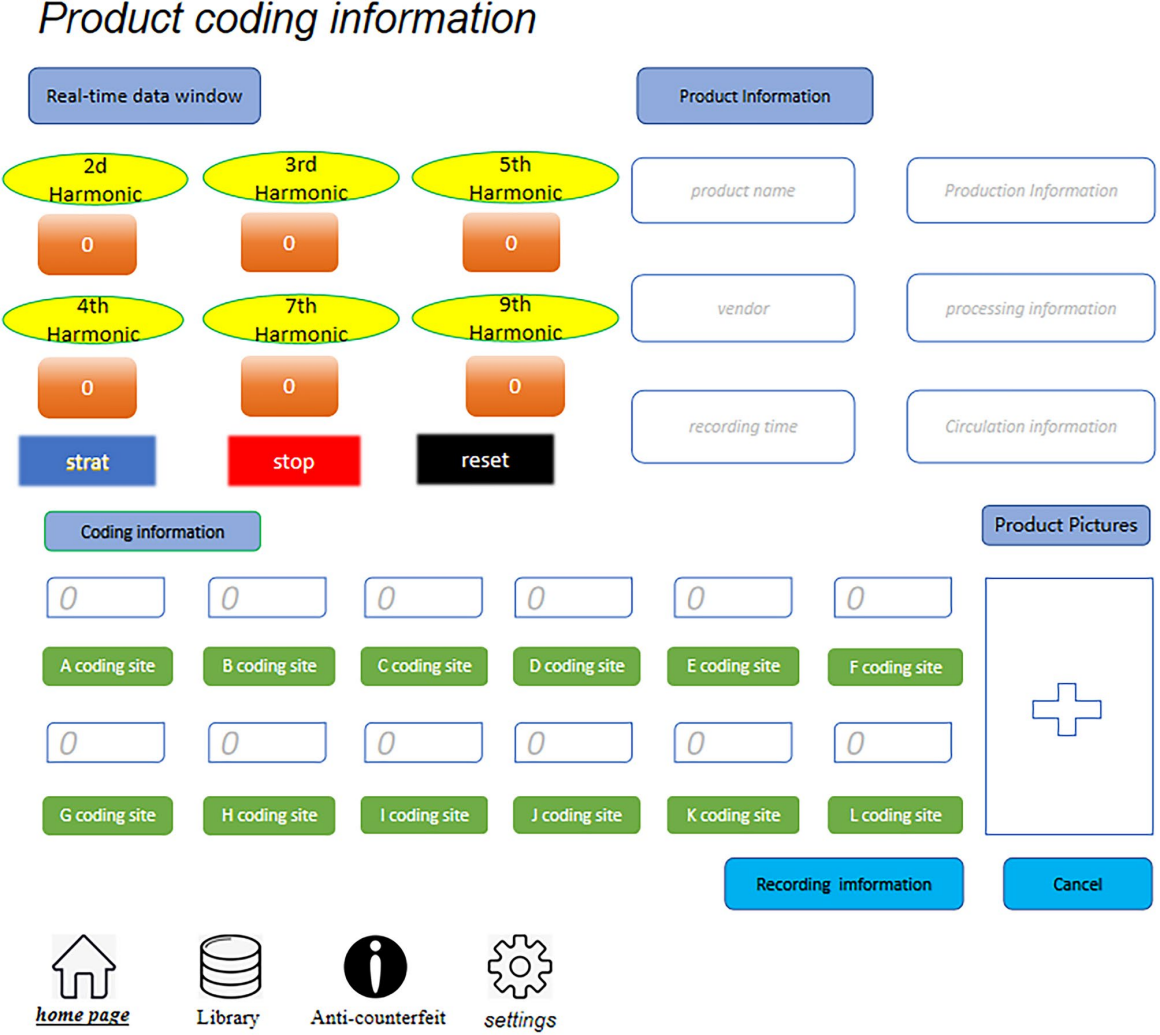
Product coding and decoding information interface diagram.

The coding sites of various types of MNPs and their corresponding odd and even harmonics signals were thoroughly investigated. Additionally, solutions containing different types of MNPs mixed at specific proportions and MNPs with functionalized surface coating were also examined. These analyses revealed distinct magnetic response signal amplitudes at each coding site. These signal values represent independent datasets without correlation. Using the coding principle and method described earlier, the number of variation parameters per code site can be multiplied to determine the number of possible coded products. The current study, the research has achieved 9000 different coded products. However, the number of products that can be coded is limited by the low number of available coding sites. To overcome this limitation, further research is needed to identify methods of increasing the number of coding data sites and expanding the number of coded products to beyond 10,000. Other approaches to enhancing coding capacity could involve optimizing the coding algorithm or improving the MNP preparation process, which may expand coding capacity without necessarily increasing the number of coding sites. Figure 9 shows the automatic coding and decoding detection system’s coding data acquisition interface.

### The stability of the interference state

It is very important for improving the practical value of our research. The effects of main environmental interferences to superparamagnetic material and its magnetic measurement including temperature, humidity and electromagnetic interference are discussed additionally.

#### Temperature effect

The magnetic properties of MNPs may be affected by the change of ambient temperature during practical application, because the superparamagnetism of MNPs is a very special property that the thermal energy exceeds the magnetic anisotropy barrier. Therefore, the temperature is a critical interference factor that may affects the stability of the developed magnetic coding and decoding system.

As the example of temperature interference, MNP sample D (0.1 mL) was diluted with 0.4mL PBS, and separated to four same sample bottles (0.1 mL diluted sample in each bottle).Four samples were heated to 25 °C, 30 °C, 35 °C and 40 °C in air heating bathtub, respectively, and then measured immediately using the developed magnetic coding and decoding system. The excitation field and frequency were fixed at 4 mT and 1 kHz, as well as the fixed humidity and electromagnetic interference for each measurement.

**Figure 10:**
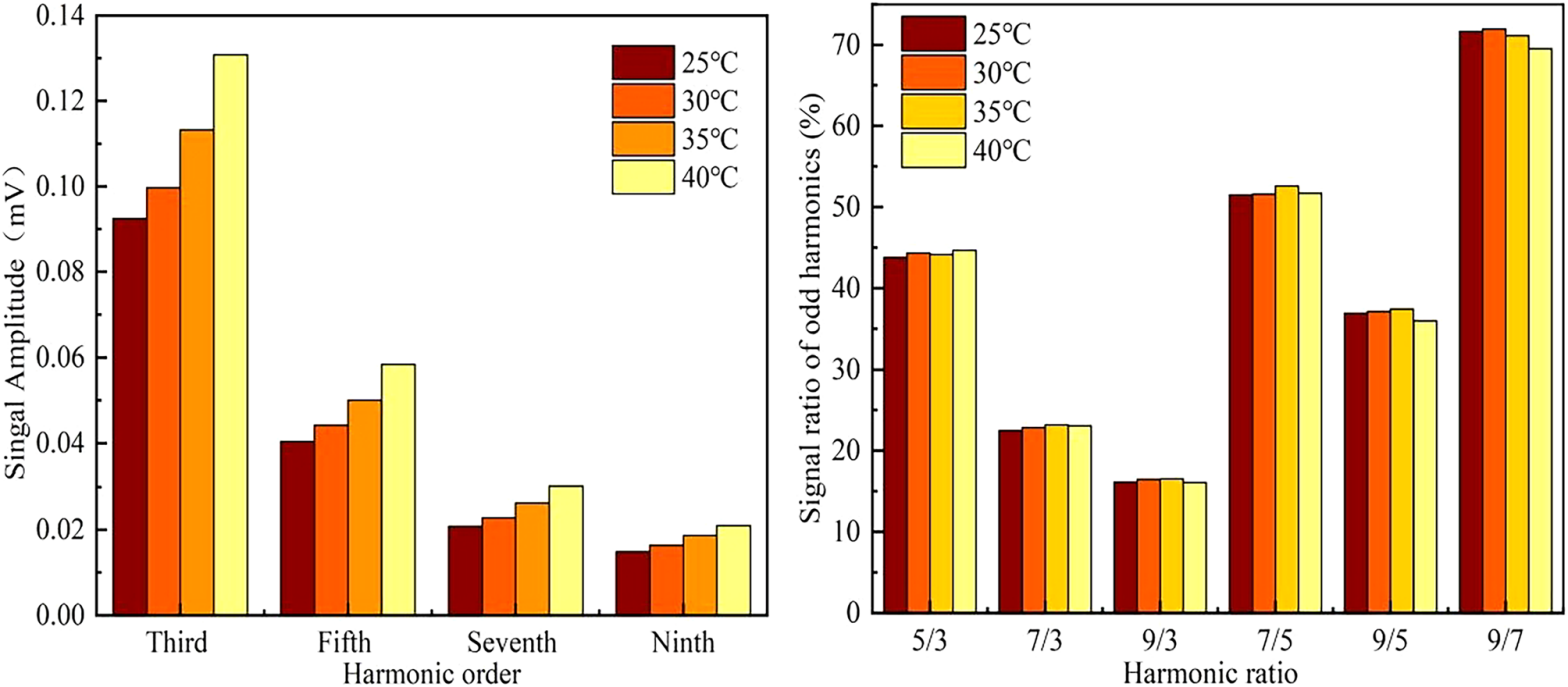
The mean amplitude of each odd harmonic and harmonic ratio of MNPs affected by environment temperature.

All sample measurements were repeated for three times, the mean amplitude of each odd harmonic and harmonic ratio were shown in Fig. 10, respectively.

The experimental results show that the harmonic response of the MNP changes with different temperatures. We think the change in harmonic amplitude was due to the higher thermal energy and faster relaxation time. Comparing to the harmonic amplitude, the harmonic ratio gives a much better stability, because the temperature variation was cancel out by proportional calculation. The maximum change in harmonic ratio was about 2% (9/7), and the average was less than 1% in all other different temperature cases. Moreover, the change in harmonic amplitude was computable and predictable due to its monotonic variation with temperature. It means we can calculate the accurate signal by introduce a simple temperature measurement and feedback system if the original harmonic amplitude signal is necessary in coding and decoding.

#### Humidity effect

**Figure 11:**
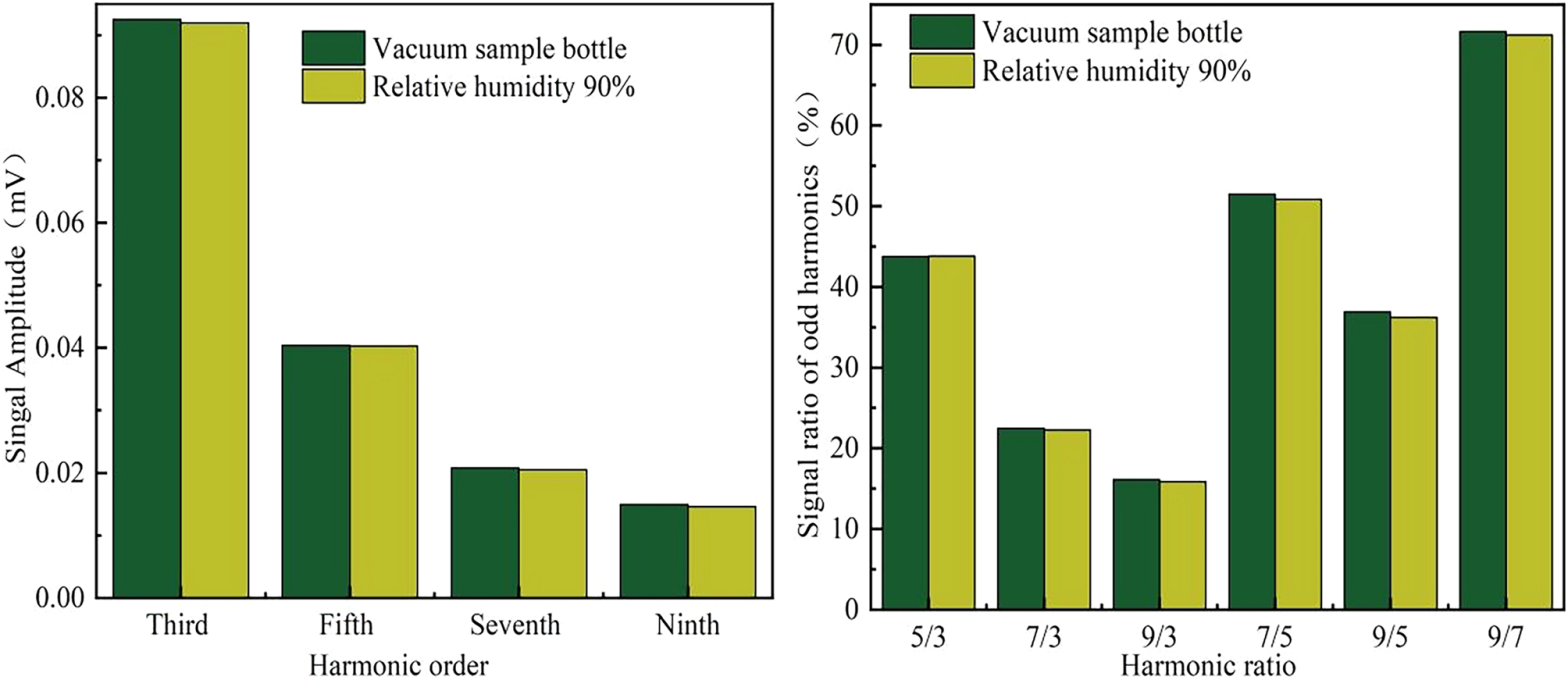
The mean amplitude of each odd harmonic and harmonic ratio of MNPs affected by environment humidity.

Humidity is another factor that may affect the properties and stability of MNPs. In the humidity interference experiment, two MNP samples were prepared in the same process of temperature case except heating. Two of the samples were placed in vacuum bottle (humidity about 0%), and other two samples were placed in 90% relative humidity environment (produced by water vapor) at room temperature. All samples were measured three times using the developed magnetic coding and decoding system (total six measurements for vacuum and 90% humidity, respectively), and the mean amplitude of each odd harmonic component and calculated harmonic ratio were shown in Fig. 11, respectively.

The experimental results show that the humidity does not affect either the harmonic amplitude or calculated harmonic ratio. We obtained good stability in severe environment humidity changes.

#### Electromagnetic interference

**Figure 12:**
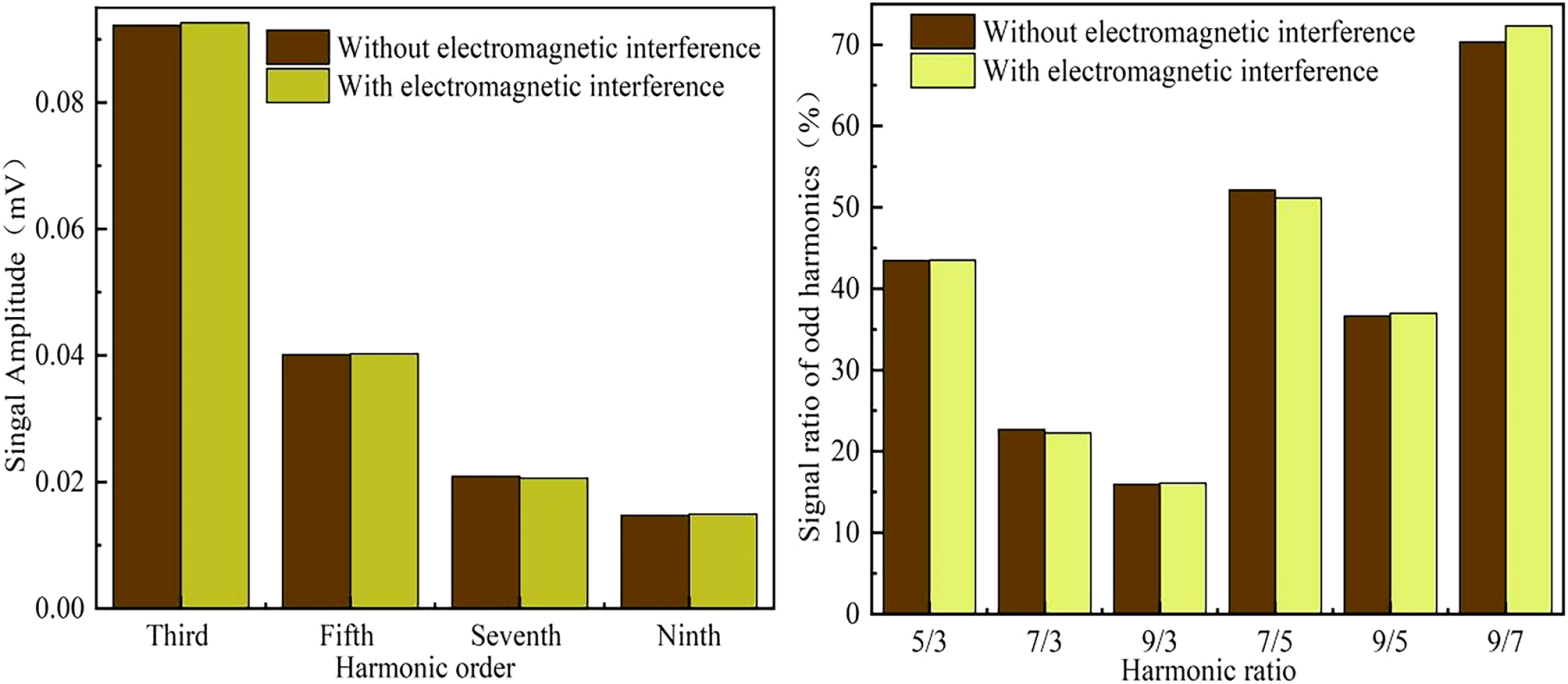
The mean amplitude of each odd harmonic and harmonic ratio of MNPs affected by environment electromagnetic.

Electromagnetic interference may affect the properties of MNPs, as well as the stability of the system. In the electromagnetic interference experiment, one MNP sample was prepared same to the temperature interference case except heating process (take one sample bottle instead of four). We thought that the main electromagnetic interference in nature environment was the power frequency (50 or 60 Hz), thus, the electromagnetic interference was produced by a pair of Helmholtz coil with a frequency of 50 Hz and a strength of 4 mT. The Helmholtz coil pair was set nearby the sample detection area, and then the mean amplitude of each odd harmonic and harmonic ratio were recorded in Fig. 12 with and without interference.

The experimental results show that the outer electromagnetic field affects the detection result very slightly. The maximum change in harmonic ratio was about 2% (9/7), and the average was less than 1% in all other cases including harmonic amplitude and ratio. We thought the slight change in harmonic ratio was mainly due to the frequency multiplication between 50 Hz (interference) and 1000 Hz (excitation field), which may cause some interference in signal to noise ratio (SNR). An adjustment of excitation frequency, such as 1000 to 1020 Hz, will be effective in further reducing of environmental electromagnetic interference. Moreover, the mixture of the low and high frequency excitation field may cause some more complex magnetization. For example, we can earn some harmonic components in 1000 ± 100 Hz and 1000 ± 200 Hz, which may contribute more encodable magnetizations. However, we can obtain good stability in severe environment electromagnetic interference.

As a shot summary, usual environmental interferences within the scope of daily life do not affect the stability of the MNPs and measurement system significantly or can be compensated easily. We think the developed magnetic coding and decoding system was suitable in a large number of daily scenes.

#### Strong magnetic field

**Figure 13:**
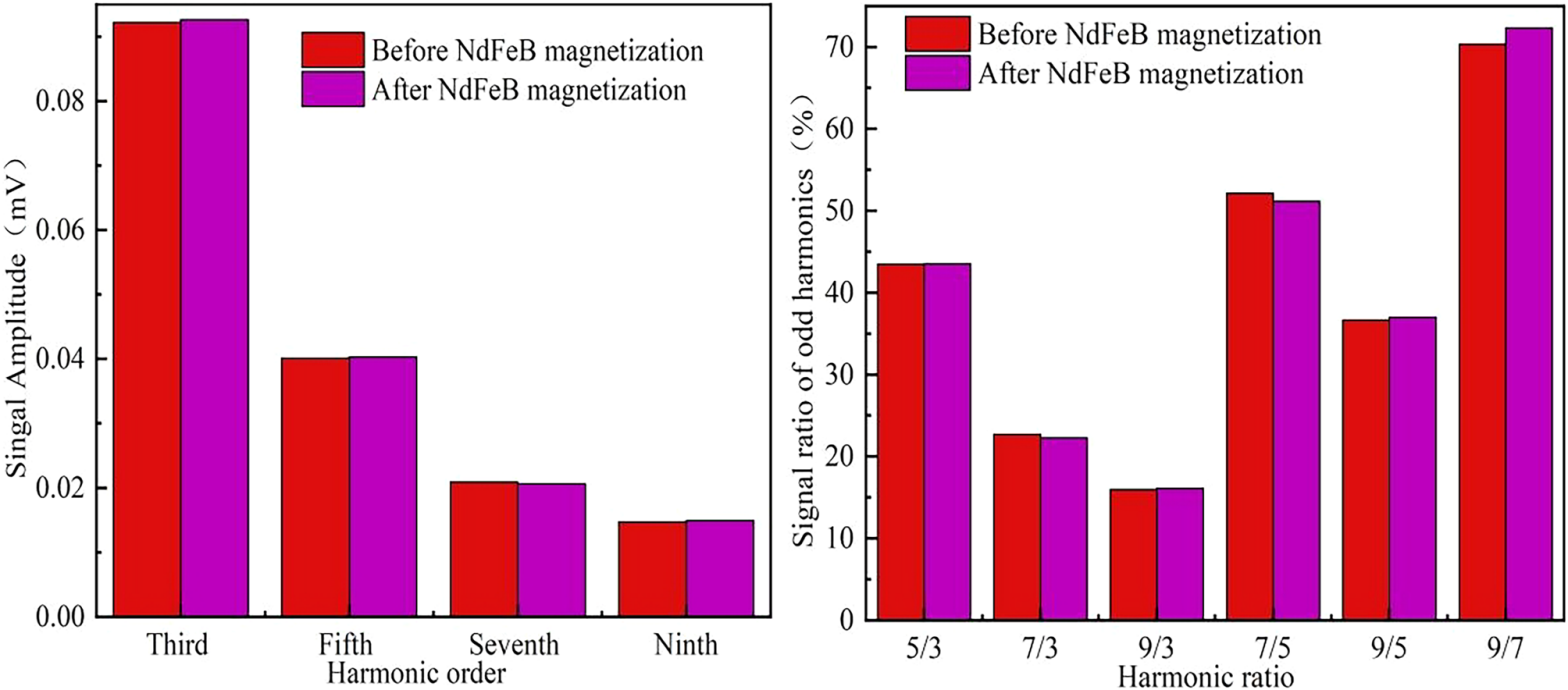
The mean amplitude of each odd harmonic and harmonic ratio of MNPs affected by NdFeB magnetization.

In theory, superparamagnetic nanoparticles should lose their magnetization due to the thermal energy and very fast Brownian or Neel relaxation time if the external magnetic field is moved out. However, there is always some small remanence in large nanoparticles with high magnetic moment that suitable for AC magnetization measurement. A typical application of this remanence is named magnetic hyperthermia. In this paper, we measured the signal stability of the MNPs before and after a strong magnetization to confirm the influence of the external magnetization that may be difficult to avoid in daily life. The magnetic interference field was produced by a NdFeB magnet with a surface magnetic field of 450 mT. 0.1 mL of MNP sample D diluted by 5 times in PBS solution was used for further experiment. The strength and frequency of the measurement excitation field was 4 mT and 1000 Hz, respectively. The MNPs sample was placed beside the NdFeB magnet for 10 min for magnetization. The odd harmonic components and calculated harmonic ratios before and after NdFeB magnetization were recorded in Fig. 13. The experiment was performed three times and the mean amplitude was final record to reduce the measurement error.

The experimental results show that the magnetization due to the NdFeB magnet affected the detection stability very slightly. The maximum change in harmonic ratio was about 2% (9/7), and the average was less than 1% in all other cases including harmonic amplitude and ratio. We thought the slight change in harmonic ratio 9/7 was due to the small harmonic response strength and measurement error but not the NdFeB magnetization, because all other measurement results were very stable. We think the stability of the MNPs sample to external magnetization was due to the small core and hydrodynamic size of the nanoparticle, but may become severe for particles larger than 30 nm core diameter. As a shot summary, we think the interference from external strong magnetic field do not affects the stability of the MNP in current developed system significantly. However, we should still avoid being too close to other magnetic fields during the measurement, as it can affect the non-linear magnetization response even saturate the MNPs sample.

### Information management

**Figure 14:**
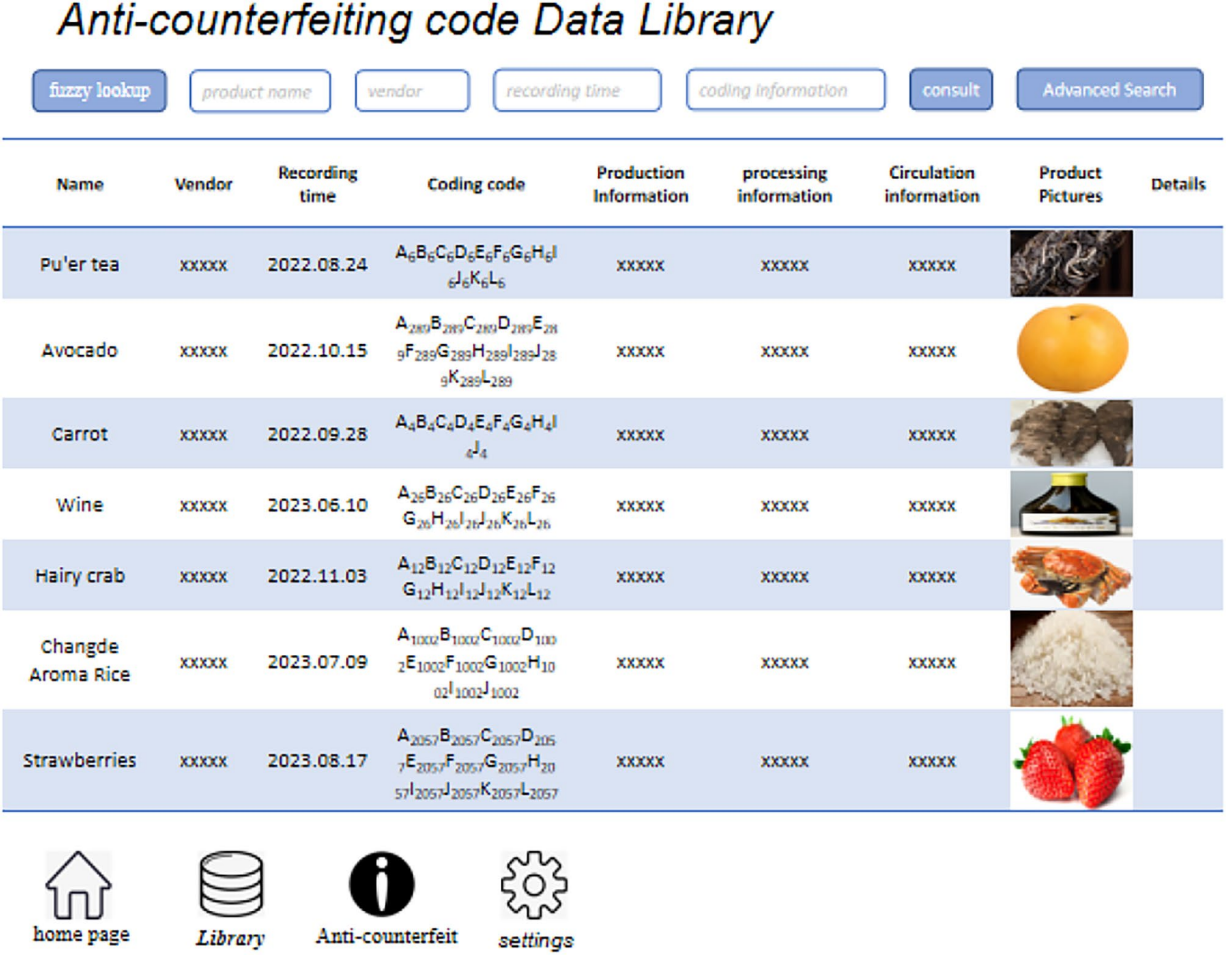
Anti-counterfeiting coding database.

Product anti-counterfeiting involves the following segments: production, processing, coding, circulation, and restoration. Production involves capturing fundamental product information related to the production process and other critical details. This information helps establish a product’s authenticity and traceability. In the processing stage, high-value-added products are preserved and packaged, and important data like the production date, batch number, location, processing details, and logistics information are recorded. These records are important for maintaining product integrity throughout the supply chain. In coding, MNPs are used in the anti-counterfeiting labels or marks added to products. Coding enhances product security and protection against counterfeiting. During circulation, coded products are transported and sold through the distribution process, with movement across various channels until they reach the end user. Restoration involves using the developed magnetic encoding and decoding system to extract coded information from the MNP-containing anti-counterfeiting marks. This information is then compared with the coding database to verify product authenticity. The anti-counterfeiting code database serves as a repository of detailed product information. As the number of coded products increases, the database is regularly updated to maintain data accuracy and completeness. Using network technology, the anti-counterfeiting code database can synchronize and share real-time data with each segment’s information systems. Consumers can scan product codes or manually enter them to access various information, including production details and distribution networks. Figure 14 illustrates the anti-counterfeiting coding database.

## Conclusion

### Detecting counterfeit products

#### Anti-counterfeiting theory

Wine anti-counterfeiting is an important task^34^. We adopted the coding method based on the MNPs studied. Using a 12-code sites and experimental data, we established the magnetic code information for wine and used the developed magnetic encoding and decoding system to measure the amplitudes of the odd and even harmonic signals of wine samples containing MNP anti-counterfeiting marks^35^. The magnetic code information of each coding site was then verified individually, measuring the magnetic response signal and verifying the coded data can accurately determine the wine’s identity and offer a strong product guarantee to consumers.

#### Anti-counterfeiting analysis

Calibration of the automatic coding and decoding detection system is a key step to ensure the accuracy of the experiment. Calibrate the excitation magnetic field intensity, eliminate the variable factors in the experiment, and ensure the uniformity of experimental conditions, repeated experiments can help confirm the stability and repeatability of the results. By calculating the mean and standard deviation of the experimental results, the consistency and stability of the experiment can be evaluated, so as to improve the reliability of the results.

Analyze the following steps to create wine anti-counterfeiting labels using MNPs.

1. Sample D (0.1 mL) was diluted in PBS (0.4 mL).
2. From the diluted sample, 0.1 mL was used to create the first anti-counterfeit label using the laser method.
3. Sample F (0.1 mL) was diluted in PBS (0.4 mL).
4. The diluted sample D (0.1 mL) was mixed thoroughly with 0.3 mL of the diluted sample F to create the second anti-counterfeiting label.
5. Sample D (0.1 mL) was diluted in PBS (0.4 mL) and 0.9% saline was prepared.
6. Sample D solution (0.1 mL) was thoroughly mixed with 0.1 mL of 0.9% saline to create the third anti-counterfeiting label.

**Figure 15:**
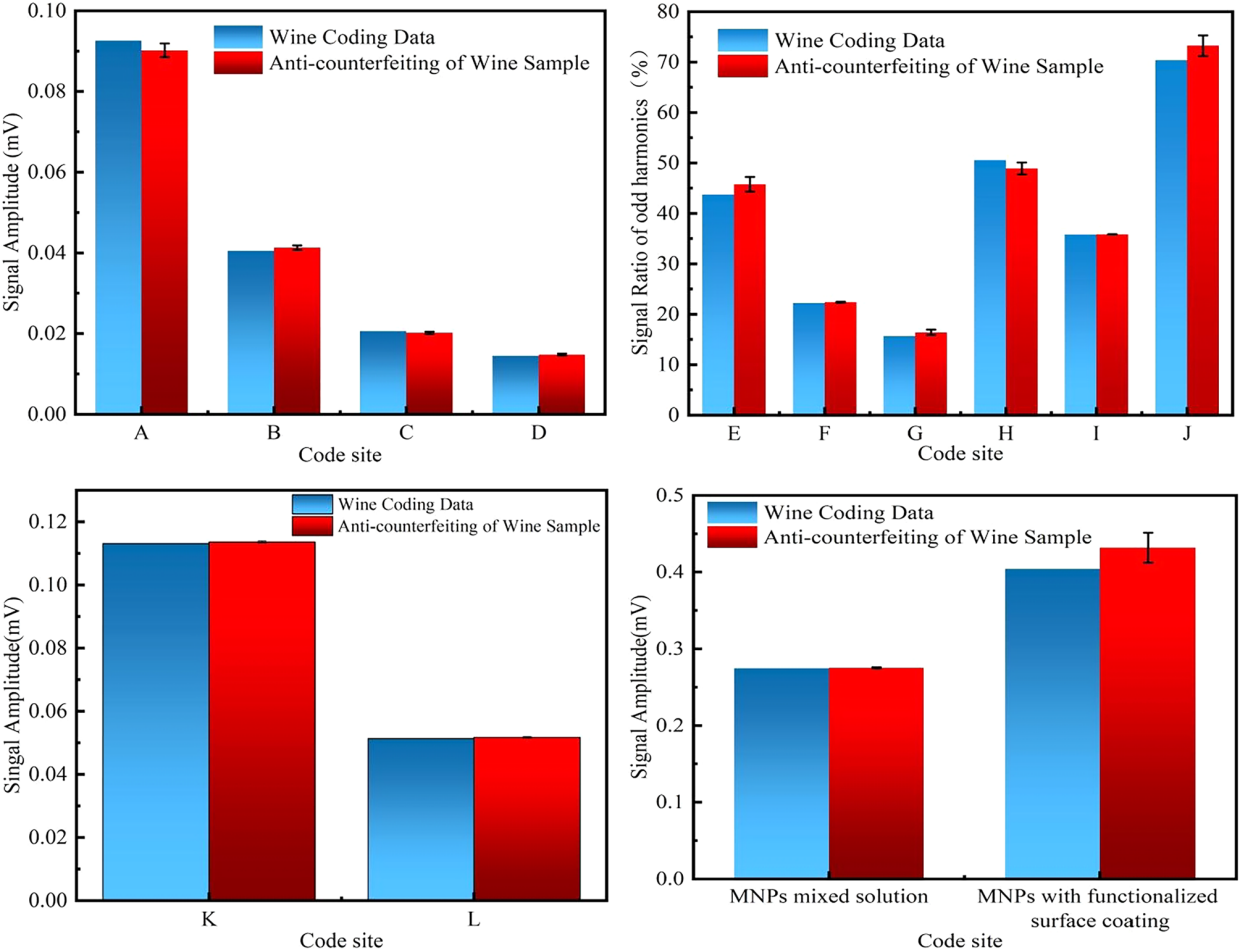
A comparison of wine coding data and wine anti-counterfeiting measurement data. The deviation range of the coded data is within 3%, indicating that the anti-counterfeiting coding technology can be used for anti-counterfeiting.

Three wine bottles available in the market were selected and the three anti-counterfeiting labels were attached to the cap opening of one wine bottle to prepare a wine sample with MNP-based anti-counterfeiting marks. It should be noted that the three bottles of wine have not yet been identified as coded products. The magnetic information of the three anti-counterfeiting labels was measured on the developed magnetic encoding and decoding system. The measured 12-digit coded data were then compared with the data in the coding database. If the deviation between the measured data and the data in the database is within ± 3%, a bottle can be verified as a coded wine sample^36^. Figure 15 shows a comparison of wine coding data and the measured wine anti-counterfeiting data.

The comparison between the wine-coded data and the sample data obtained in this study revealed a deviation of within 3%, indicating that the anti-counterfeiting method based on the magnetic coding system can effectively secure high-value-added products. The developed magnetic encoding and decoding system exhibited the ability to accurately detect the magnetic information embedded in the anti-counterfeiting labels. Because of its several advantages, including uniqueness, simplicity, stability, and security, the coding system can be widely used to code and recognize various products. By following unified coding rules, this system can address the challenges faced by manufacturers and organizations when implementing their coding systems, since it facilitates management, product traceability, and information exchange throughout the industrial chain process. This has significant implications for the improvement of production efficiency and safeguarding consumer rights and interests.

#### Comparative analysis

Traditional anti-counterfeiting methods mainly use stable isotopes to achieve product traceability. Due to the extremely low content of stable isotopes, their content is usually detected by mass spectrometers. Table 6 shows the mass spectrometer test process^37,38^. Superparamagnetic nanoparticles are used as high performance coding materials to judge the authenticity of the samples by the self-developed automatic coding and decoding recognition system. Table 7 shows the detection process based on superparamagnetic nanomaterials coding technology.

**Table 6:** Mass spectrometer test process.

Detection procedure	Step description	Detection time
Sample preparation	Sample collection, pretreatment, decomposition, extraction, etc	More than 10min
Sample injection	It is injected into the mass spectrometer in gaseous, liquid or solid form	
Ionize	The molecules or atoms in the sample are ionized, in the process of ionization, the molecules or atoms in the sample lose or gain electrons, forming charged ions	
Mass spectrometry	The test is carried out in the detector of the mass spectrometer	
Data analysis	The resulting data is presented in the form of mass spectrograms	
Calibration and calibration	Calibration and calibration of mass spectrometers	
Report results	Reported as isotopic content or isotopic ratio

**Table 7:** The detection process based on superparamagnetic nanomaterials coding technology.

Detection procedure	Step description	Detection time
Sample preparation	Place the product under test with the magnetic nanoparticle label into the testing instrument	1 min
Product testing	The magnetic information of the magnetic nanoparticle material is automatically detected and analyzed by the detector based on the 12-site coded data	
Report results	Reports in the form of testing the authenticity of products	

As can be seen from Tables 6 and 7, the use of superparamagnetic nanoparticles as high-performance coding materials has more advantages than traditional isotope detection methods in terms of detection process and detection time, and the detection process does not require professional personnel to operate.

In terms of cost, the price of isotope mass spectrometers varies by model, brand, performance, and configuration, ranging from tens of thousands of dollars to millions of dollars. The superparamagnetic nanomaterial coding technology is relatively economical, and the detection cost of each product to be tested is within $1. In addition to the daily maintenance of the instrument, the isotope mass spectrometer also needs to consider the maintenance cost of the peripheral part, and the maintenance cost of the automatic encoding and decoding recognition system based on superparamagnetic nanomaterials is mainly focused on the daily maintenance and use of the instrument itself, and the cost is relatively low. Automatic coding and decoding detection systems based on superparamagnetic nanomaterials do not require additional training and are relatively simple to operate, while isotopic mass spectrometry rules require systematic training, which may add additional cost and time investment.

The encoding technology based on superparamagnetic nanoparticle materials uses the magnetic properties of magnetic nanoparticles to store information, and realizes the storage of single or multi-value information by adjusting the magnetic moment size, direction and other parameters. Due to the small size and high information density of magnetic nanoparticles, efficient information storage and management can be achieved at the micro scale. The combination of particle size, shape and magnetic properties is flexible to meet different product coding requirements, so its storage capacity and performance are relatively high. In contrast, stable isotope coding takes advantage of the special properties of isotopes. Although 274 stable isotopes have been found, the main applications are still a few, such as deuterium, carbon-13, nitrogen-15 and so on. As a result, its storage capacity is relatively low due to isotope type and quantity limitations.

In general, superparamagnetic nanoparticle coding technology has significant advantages in terms of information storage capacity and flexibility, as well as maintenance costs and personnel training.

### Future directions

The coding technique based on superparamagnetic nanomaterials proposed in this paper is applied to other industries. For example, in the pharmaceutical industry, magnetic nanoparticles are added to drugs to ensure the authenticity and safety of drugs, prevent counterfeit drugs from entering the market, and ensure the safety of patients. In the food industry, by adding magnetic nanoparticle materials to food packaging or labels, food information can be recorded and traced to improve food safety. In the retail industry, the realization of smart retail and personalized recommendations can enhance the consumer shopping experience by scanning the magnetic nanoparticle encoded information of products. In the agricultural products industry, an information traceability platform based on magnetic nanoparticle coding can be established to achieve the entire information record and query from production to sales, and improve the quality and safety of agricultural products. It can also be combined with artificial intelligence and big data technology to achieve intelligent management and data analysis of coded products, and improve the anti-counterfeiting effect and management level^39,40^.

## Data Availability

The datasets generated and/or analysed during the current study are either included in the published article itself (or available from the corresponding author upon reasonable request). The datasets on the submission system are available in the article (or available from the corresponding author upon reasonable request).
